# A Psychometric Perspective on the Associations between Response Accuracy and Response Time Residuals

**DOI:** 10.3390/jintelligence12080074

**Published:** 2024-07-31

**Authors:** Weicong Lyu, Daniel Bolt

**Affiliations:** 1 College of Education, University of Washington, Seattle, WA 98105, USA; 2 Department of Educational Psychology, University of Wisconsin, Madison, WI 53706, USA

**Keywords:** response time, item response theory, response mixture analysis

## Abstract

We provide an alternative psychometric perspective on the empirical statistical dependencies observed between response accuracy residuals (RARs) and response time residuals (RTRs) in the context of the van der Linden model. This perspective emphasizes the RAR (or parts of the RAR) as being exogenous and having a directional influence on response time. Our simple and theoretically justifiable perspective adds to previous joint response time/accuracy models and comports with recent generalizations of the D-diffusion IRT model incorporating person-by-item interactions, and thus similarly reproduces many of the recently highlighted empirical findings concerning the associations between RARs and RTRs. Using both empirical and simulation-based results, we show how our psychometric perspective has both applied and interpretational implications. Specifically, it would suggest that (1) studies of item parameter estimate heterogeneity in relation to response times may reflect more of a psychometric artifact (due to the exogenous effects of the RARs) as opposed to providing insights about the response process (e.g., the application of different response strategies) and that (2) efforts to use RTRs as indicators of latent proficiency should attend to the anticipated interactions between the latent proficiency and RAR on response times. The validity of our psychometric perspective against alternatives likely relies on appeals to theory; the best perspective to take may vary depending on the test setting.

## 1. Introduction

Response times reflect are a potentially useful source of data that can be used for various purposes in cognitive and noncognitive tests. For tests of cognitive proficiency, much recent attention has been given to aspects of van der Linden’s (2007) joint hierarchical model for response accuracy and time that may need to be generalized to allow the model to better accommodate response accuracy–time statistical dependencies. For each person x item response, van der Linden’s (2007) model provides a joint representation of their response accuracy and response time in relation to both person parameters (e.g., person proficiency, person speed parameters) and item parameters (e.g., item difficulty, item discrimination parameters for response accuracy, item time intensity, item discrimination for response time), with corresponding multivariate distributions within person/item parameter types. As van der Linden’s model implies conditional independence between response accuracy and response time once accounting for such parameters, recent attention has focused on the empirical patterns of statistical dependence commonly seen between the *response accuracy residuals* (RARs) and *response time residuals* (RTRs) in this model (see, e.g., De Boeck and Rijmen 2020). Understanding and accounting for such associations is important in allowing the joint model to function more effectively in allowing response times to possibly inform the measurement of the latent proficiency (Bolsinova and Tijmstra 2018). However, these dependencies have also been viewed as informative about aspects of the psychological response processes underlying test items, including how within-respondent variability in speed across items might correspond to the adoption of different strategies, strategies that have different implications for response accuracy outcomes, for example (e.g., Molenaar and De Boeck 2018). Most interpretations of RAR-RTR associations in the literature have emphasized issues of interest in mathematical psychology, such as the speed–accuracy tradeoff, and consequently focus on how intentional variations in response speed may have implications for accuracy. Our goal in this paper is to present an alternative psychometric perspective that emphasizes the response accuracy residual as an influence on response time and to discuss the implications of this alternative perspective for interpretations.

### 1.1. Explaining RAR/RTR Statistical Dependencies

Bolsinova et al. (2017a) provided a useful overview of different perspectives that can be adopted in understanding the relationships between RARs and RTRs. As we seek to show in this paper, an additional and theoretically justifiable perspective to those they present emphasizes components of the RARs as exogenous predictors of response time. This may at first seem an unusual perspective, as the score a respondent receives on an item seemingly follows the time spent working on the item, and thus if any causal directionality is assumed, we might reflexively view response time as predicting response accuracy as opposed to the opposite. Indeed, the only model presented by Bolsinova et al. (2017a) that introduces a directional relationship between RTRs and RARs implies that the RTR influences the RAR. However, in the context of many educational and cognitive tests, we suggest the reverse directionality may be reasonable and could in fact be expected. Specifically, we might view components of the RAR (e.g., the latent specific knowledge of a respondent regarding a particular item that is separate from their latent proficiency) as reflecting characteristics of the respondent that were brought to the experience of the item and thus are exogenous predictors of not only response accuracy, but also response time. Such characteristics could reflect aspects of conceptual, procedural or other forms of knowledge that the respondent has that are specific to the item and that determine response accuracy.

As an example of how such a component might include elements of conceptual knowledge, consider, for example, a conceptual math test item that we assume, for illustration, is of moderate difficulty: “What is the sum of the angular degrees across angles within a triangle?”. We can imagine one respondent who is high on the general latent proficiency variable (e.g., math ability) but who (prior to the test) did not know the concept that would eventually be tested by this item. We can imagine another respondent who is moderate on the latent proficiency variable but who (prior to the test) definitely knows this concept. From our alternative perspective, the high-proficiency respondent would be understood as having a *low* expected value on the RAR for this item, while the moderate-ability respondent would have a *high* expected value on the RAR for this item. Viewing this component of the RAR as a type of item-specific latent person trait (albeit not an estimable one) allows us to appreciate the better expected performance on this item of the moderate-proficiency respondent relative to the high-proficiency respondent.

As an example of an item where such a component might reflect an element of procedural knowledge, consider the math test item “$\frac{4}{7}-\frac{1}{8}=?$”, where knowledge about determining a common denominator will be important to obtaining a correct item response. Along the same lines as the previous example, we could imagine a respondent who is low on overall math ability that knows how to do this and another respondent moderate on overall math ability that does not. Similar to the previous example, even though there is more than just knowledge about determining a common denominator involved in this item, we might still consider the hypothetical low-ability respondent as possibly having a higher expected score on the item than the moderate-ability respondent. It is likely that the moderate-ability respondent will struggle more on the item than the low-ability respondent.

In both examples, we can appreciate the trait-like component of the RAR as an aspect of the respondent that was brought to their encounter with the item and thus better viewed as exogenous. Of course, as illustrated by the second example, there may be other elements of the RAR that emerge at the time of item exposure (e.g., a student identifies the common denominator, but miscalculates the corresponding adjustment to one of the numerators, or miscalculates the difference in the numerators). However, if we appreciate that the trait-like part of the RAR may function in interchangeable ways with general latent proficiency in its effects on both response accuracy AND response time, the role of the RAR as having a directional influence on response time becomes plausible.

### 1.2. Attending to Item-Specific Factors in a Conceptual Psychometric Model

The perspective in the previous section that views aspects of the RAR as exogenous respondent characteristics specific to the item was presented by Lyu et al. (2023) in the context of wide-ranging applications of sequential IRT and IRTree models. It is also a perspective that comports with Kang et al.’s (2022) concept of person–item interactions in the diffusion IRT model, as this component of the RAR can be viewed as a person x item interaction. As a result, in a similar way to Kang et al. (2022), this perspective can help us understand many of the empirical observations seen in the literature that examine relationships between response accuracy and response time. Lyu et al. (2023) consider such person x item interactions as *item-specific factors*, a concept that is not new but historically a part of how a common factor and residual variance are understood in the common factor model (e.g., Harman 1967). Specifically, a variable’s residual in the common factor model is understood to be a confounded combination of variance specific to the variable (e.g., a “specific factor”) and error. Arguably, in a response time analysis, we might well expect the item-specific factor and respondent’s proficiency to function in equivalent ways in affecting response time. In the context of a simulation illustration, we seek to illustrate the implications of this perspective for interpreting item parameter heterogeneity in relation to response time and in explaining the empirical patterns in the dependence between RARs and RTRs.

## 2. Materials and Methods

### 2.1. Distinguishing the General and Item-Specific Components of Proficiency Underlying Response Accuracy

In this paper, we use θ to denote the latent general proficiency of the respondent and $\eta_{i}$ to denote the exogenous trait-like part of the RAR (i.e., item-specific factor) for item i on a multi-item test. We consider $\eta_{i}$ as at least part (and possibly all) of the RAR that is, by definition, orthogonal to θ. Further, as part of the RAR, $\eta_{i}$ combines with θ in an additive way to predict the accuracy of the response. As presented by Lyu et al. (2023), we then think of the response accuracy model of respondent h to item i as a conceptual model in which the probability of correct response is written as

(1) $$P\left(U_{hi}=1|\theta_{h},\eta_{hi}\right)=P\left(\theta_{h}+\eta_{hi}-b_{i}+\varepsilon_{hi}>0|\theta_{h},\eta_{hi}\right),$$

where $U_{hi}=1$ denotes a correct response, $b_{i}$ denotes the IRT difficulty of the item, and $\varepsilon_{hi}$ is a zero-mean random variable following a logistic distribution for logistic models and a normal distribution for normal ogive models. This conceptual model can be generalized by adding item discrimination parameters, a multidimensional θ, etc., but in the current setting the representation in (1) is sufficient for our purposes. In effect, for a given item *i*, we have two relevant latent person parameters, θ and $\eta_{i}$, where $\eta_{i}$ can be viewed as a similarly scaled person trait that reflects a respondent’s interaction with item i. (Note that, in some instances, specifically when emphasizing θ and $\eta_{i}$ levels for a particular respondent h, we include h as a subscript on these parameters; however, in instances when we are talking about these features more generally, we omit the h subscripts.) As implied in (1), both θ and $\eta_{i}$ are assumed to have a consistently positive effect on response accuracy. Essentially, we are just replacing θ in our usual response accuracy model with ${\theta +\eta }_{i}$ to emphasize our belief that a part of the response accuracy residual can also be viewed as a latent person trait, albeit one that is specific to item i (e.g., item-specific knowledge).

### 2.2. Relating the General and Item-Specific Components of Proficiency to Response Times

For response times in time-limited tests (or in non-time-limited tests where the time spent on the test may still be a valued resource), it can often be suspected that faster response times on an item reflect two very different causes—(1) a respondent who clearly knows how to answer/solve the item and can report the correct answer quickly and (2) a respondent who clearly does not know how to answer/solve the item and chooses to move past the item (perhaps making a somewhat quick, more likely incorrect, guess). Both of these instances can be understood in relation to θ, the latent proficiency of the respondent. The combination of these two conditions can lead to a type of inverse U or inverse V relationship between the respondent’s expected performance on the item (typically viewed as a function of their general latent proficiency and the item parameters, such as difficulty) and response time. Respondents taking the longest time are those for whom their expected performance on the item is in between the extremes of the two levels of expected performance, such as a respondent that has a 0.5 probability of correct response. Such respondents might be expected to be sufficiently engaged with the item to continue working on it, but not so certain of the correct response as to be able to provide the answer quickly. If we appreciate $\eta_{i}$ as functioning in interchangeable ways with general latent proficiency in regard to expected response accuracy, it can be appreciated from the above theory that θ and $\eta_{i}$ should also be interchangeable in their influence on the response time for item i. For our two hypothetical respondents (the medium-proficiency respondent who knows the answer and the high-proficiency respondent who is uncertain), we expect a faster response time for the medium-proficiency respondent on this item (despite a general latent proficiency that would imply a slower response), but a slower response time from the high-proficiency respondent. Such a perspective appears consistent with the anticipated response times in the presence of explicit scoring rules that are sensitive to response accuracy and time (Maris and Van der Maas 2012). Moreover, even for tests without such explicit scoring rules, but for which the speed of response is implicitly rewarded (e.g., by the ability to devote more time to other items, by the ability to finish the test early) we might anticipate a similar functional form. Such conditions might be viewed as reflecting a combination of both spontaneous and imposed speed (see, e.g., De Boeck et al. 2017) within the same test. Related observations are also seen in measures of noncognitive traits using rating scale instruments, where the slowest responses are often seen for respondent–item interactions that lead to expected responses in the middle of the rating scale, and faster times are seen as the expectation is away from the middle (Ranger 2013; Ranger and Ortner 2011; Kim and Bolt 2023).

### 2.3. Empirical Examination of Response Time against Proficiency

**Figure 1:**
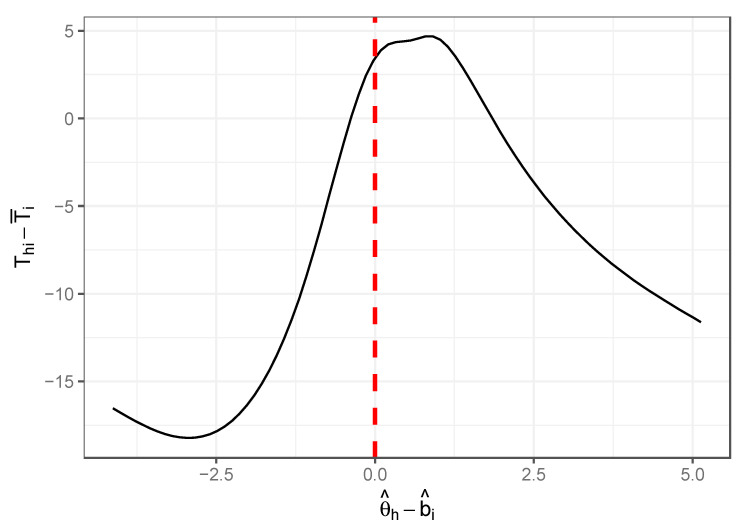
Average estimated relationship between latent proficiency, item difficulty difference, and response time over 75 mathematics test items on a college placement test.

As an empirical illustration of this relationship in a cognitive achievement test, we used response time and accuracy data for 5929 respondents to a 75-item test of mathematics used for college placement at a university in the midwestern United States. We fit a Rasch model to the item responses to obtain estimates of both person trait ${\hat{\theta }}_{h}$ and item difficulty ${\hat{b}}_{i}$ for each respondent and item. For each item i, we computed its mean response time ${\bar{T}}_{i}$ across all respondents. Figure 1 shows the nonparametric regression curve of the demeaned response time ${T_{hi}-\bar{T}}_{i}$ against ${\hat{\theta }}_{h}-{\hat{b}}_{i}$ across all responses. It clearly shows an inverted U/V relationship with a turning point near zero, suggesting that response times are longest when the probability of answering the item correctly is near 0.5. As responses associated with a ${\hat{\theta }}_{h}-{\hat{b}}_{i}$ below 0 are more likely incorrect responses, and those associated with a ${\hat{\theta }}_{h}-{\hat{b}}_{i}$ above 0 are more likely correct responses, our speculation that respondents appear to put more time into items where time is needed and useful appears to be a reasonable assumption.

**Figure 2:**
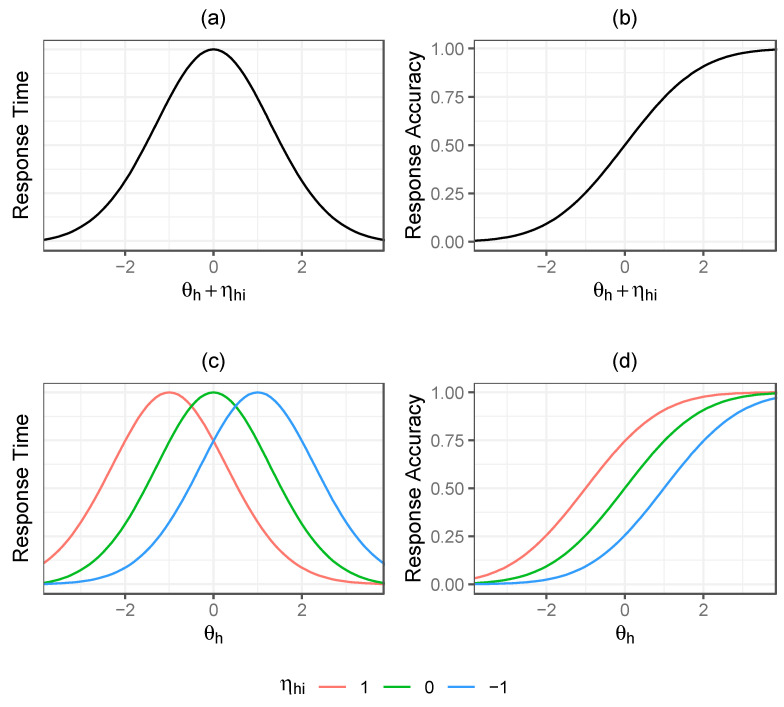
Theorized relationships for a hypothetical item of medium difficulty ($b_{i}=0$), characterizing (**a**) response time (RT) against general latent proficiency ($\theta_{h}$) + item-specific factor ($\eta_{hi}$), (**b**) response accuracy (RA) against general latent proficiency ($\theta_{h}$) + item-specific factor ($\eta_{hi}$), (**c**) response time (RT) against proficiency ($\theta_{h}$) for example levels of the item-specific factor ($\eta_{hi}$), and (**d**) response accuracy (RA) against proficiency ($\theta_{h}$) for example levels of the item-specific factor ($\eta_{hi}$).

Figure 2 provides a graphical depiction of how our psychometric model emphasizes an exogenous component of the response accuracy residual and its perceived relevance to both response time and response accuracy. The figures show theoretically how we can think about response time (RT) and response accuracy (RA) when considering the influence of both general latent proficiency and item-specific factors. In both models, the latent general proficiency and item-specific factor combine additively to influence response accuracy and response time in place of where we might traditionally only view the general latent proficiency as functioning. Importantly, while accuracy increases monotonically with the combined latent general proficiency and item-specific factor, for response time we have the inverted U/V relationship. This implies an interaction between latent general proficiency and item-specific factors on response time. If the latent general proficiency is high, the response times for a moderate difficulty item will begin increasing as $\eta_{i}$ moves away from 0 in a negative direction and decrease as $\eta_{i}$ moves away from 0 in a positive direction, while, if the latent general proficiency is low, the opposite effect occurs.

### 2.4. Resulting Psychometric Model Explaining RAR/RTR Dependence

**Figure 3:**
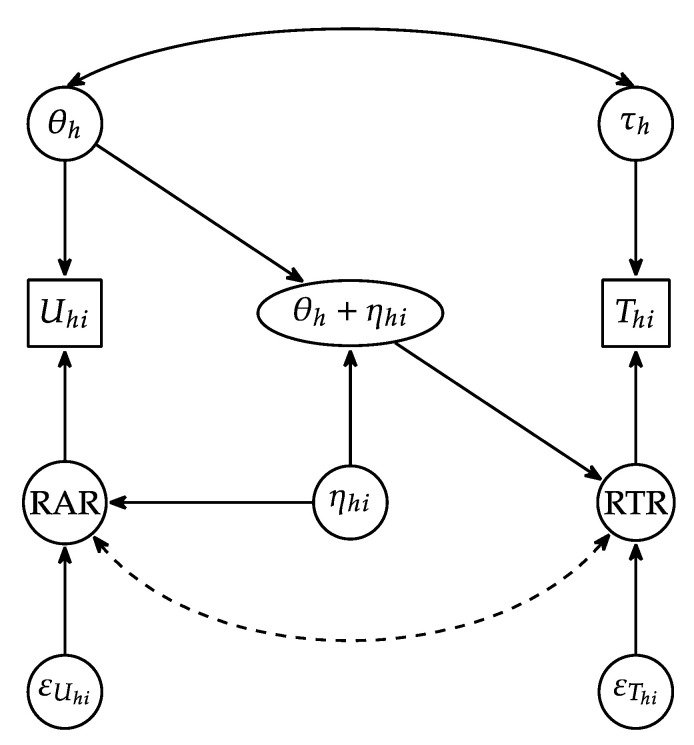
Graphical representation of the theorized relationships between general latent proficiency (θ) and item-specific factors ($\eta_{hi}$) and response accuracy ($U_{hi}$) and response time ($T_{hi}$) outcomes. The dashed curve represents the original RAR-RTR dependency being explained by the item-specific factor.

As mentioned earlier, our perspective on the RAR/RTR statistical dependencies in the joint response accuracy/response time model is an addition to the models presented in Figure 1 of Bolsinova et al. (2017a). A graphical depiction of our conceptual model for response time is shown in Figure 3. Similar to other joint accuracy and response time models, we include observations of response accuracy (U) and response time (T), as well as the underlying respondent latent traits of general proficiency (θ) and speed (τ). Also present are residuals associated with response time (RTR) and response accuracy (RAR), which we separate into two parts—a trait-like item-specific factor ($\eta_{i}$) that influences both the RAR and RTR and residual parts that are independent ($\varepsilon_{U_{i}},\;\varepsilon_{T_{i}}$). The RAR is defined as the combination of the components $\eta_{i}+\varepsilon_{U_{i}}$. Under our approach, we view the latent proficiency and item-specific factor as influencing the RTR and, more specifically, displaying a *disordinal interaction* in their predictive effects. Such an interaction is consistent with the representation in the right panels of Figure 2. Although Figure 3 makes it clear that $\eta_{i}$ need not strictly be viewed as a part of the RAR, we choose to interpret it that way, as such factors have historically been viewed as present in psychometrics (e.g., the common factor model).

As noted, our perspective on the presence of a person-by-item interaction, here represented by $\eta_{i}$, is consistent with the introduction of person-by-item interactions into the general D-diffusion IRT model (Kang et al. 2022), although now from a psychometric perspective that the interaction is an item-specific factor associated with response accuracy. Below, we use a simple simulation illustration to demonstrate these consistencies. We focus on empirical observations from two types of analyses: (1) response mixture analyses, which seek to characterize how IRT item parameter estimates change in relation to response time, and (2) empirical studies of RAR/RTR associations, as evaluated in the context of van der Linden’s (2007) model.

### 2.5. Simulation Illustration

In our simulation, we assume a response accuracy model as in (1) for 10,000 examinees completing 200 items to help make the systematic effects apparent. For simplicity and to follow (1), we assume no variability in item discrimination (i.e., a=1 for all items) and that b∼U(−3,1); in terms of the examinee parameters, we assume $\left(\theta ,\eta \right)∼N(0,I)$, where I is the identity matrix. As there is a separate $\eta_{i}$ for each item, I is a 201 × 201 matrix and 0 is a 201-element zero vector. Note that, in this simulation, the θ and $\eta_{i}$ parameters all have the same variance equal to 1. Assuming a variance for $\eta_{i}$ that is as large as the variance of θ is reasonably realistic, as the residual variance in the response accuracy score within an IRT model is generally greater (often much greater) than the variance explained by the latent general factor (Lyu et al. 2023). Using the simulation parameters generated as described above, response accuracy ($U_{i}$) is simulated as binary following

$$U_{hi}=1\left(\theta_{h}+\eta_{hi}-b_{i}+\varepsilon_{U_{hi}}>0\right),$$

where $\varepsilon_{U_{hi}}$ is generated as $N\left(0,\frac{\pi^{2}}{3}-1\right)$. We fix the variances of $\eta_{hi}$ and $\varepsilon_{U_{hi}}$ such that they sum to $\frac{\pi^{2}}{3}$ to mimic the residual of a conventional logistic model without item-specific factors (see, e.g., Norton and Dowd 2018, p. 864).

As the purpose of this illustration is only to illustrate the nature of effects on response time residuals under our alternative modeling perspective, we simplify our approach to generating response times so as to make the systematic effects of the RAR on response times more apparent. Specifically, in reference to Figure 3, we omit from our response time generation model the variability related to τ, we omit additional systematic item effects related to time, and we also omit additional response time residuals ($\varepsilon_{T}$) beyond what is accounted for by $\theta_{h}+\eta_{hi}$. These features are usually a part of empirical models attending jointly to response accuracy and response time, but only contribute what can be understood as additional variability to the response time residual distributions. These factors need not be introduced to our simulation, as our goal is simply to demonstrate the systematic effects that emerge in associations between RARs and RTRs (but see the Supplementary Materials Part I for an additional simulation example that includes such systematic effects).

As a result of this simplification, we can generate the equivalent of the response times directly in relation to ${\theta +\eta }_{i}-b_{i}$, such that the respondent’s response times are greatest when $P\left(U_{i}=1|\theta +\eta_{i}-b_{i}\right)=0.5$ and decrease linearly as $\left|P\left(U_{i}=1|\theta +\eta_{i}-b_{i}\right)-0.5\right|$ increases:

(2) $$T_{hi}=C-\left|P\left(U_{hi}=1|\theta_{h}+\eta_{hi}-b_{i}\right)-0.5\right|,$$

where C=1 is an arbitrarily chosen constant. To appropriately view these as residuals (in which both the respondent and item’s main effects have been removed), these generated values can be double-centered such that the mean value for each respondent and item is 0. What we display as the RTR in subsequent analyses are thus these double-centered values of $T_{hi}$. Like the response accuracy model, this model can be generalized in additional ways, but since the Ts will only be used to illustrate the effect patterns seen with empirical data, such generalizations are unnecessary for our demonstration. Note that (2) implies an inverted V (as opposed to inverted U) in the generation of data.

### 2.6. ApplyingResponse Mixture Analysis to the Simulated Data

Our psychometric model provides a useful alternative perspective on analyses that have demonstrated empirical relationships between response time and item characteristics. One approach that has been used to understand how response times may be associated with different psychological response processes is the *response mixture analysis* (Molenaar and De Boeck 2018). While different variations of this approach can be applied, the basic idea is to study how item response theory (IRT) parameter estimates vary for the same item in relation to fast versus slow responses. This perspective is also consistent with Model D of Figure 1 in Bolsinova et al. (2017a), whereby item-specific classes defined simultaneously by item-specific response time and accuracy differences are present. Empirical results based on such methods generally return consistent findings: in comparison to slower responses, faster responses display (1) increased item discrimination estimates which are often combined with (2) increased difficulty for difficult items and reduced difficulty for easier items (Molenaar and De Boeck 2018; Bolsinova et al. 2017b; DiTrapani et al. 2016; Partchev and De Boeck 2012; Kim and Bolt 2023). Although various interpretations have been given to these effects, they tend to emphasize the respondent as either selecting a response speed or, alternatively, a way of responding (e.g., a problem-solving strategy) that impacts both response speed and accuracy.

Following the generation of our simulated response accuracy and response time data above, we apply within-respondent median centering to the Ts of our generated response time matrix. We categorize the individual item responses as fast if the respondent-centered response time is negative, and as slow if the centered response time is positive. This approach was observed to be successful in creating response time classes where, for each respondent and for each item, half of the responses are in the fast class and half in the slow class. The item response accuracy data for both fast and slow response classes can then be concatenated by row so that each row corresponds to the same respondent and the fast versus slow responses for the same item are treated as responses to different items. This results in a data matrix in which half of the responses are missing, but where half of the responses from each respondent are in the fast and slow classes. Note that the item parameter estimates for the fast and slow response classes will be on the same scale (without any need for additional linking) due to the presence of examinees having the same θ across classes. We then fit the generated data through one 2PL calibration using ltm (Rizopoulos 2007).

## 3. Results

The results of our simulation analyses are presented in two parts. First, we demonstrate the simulation results from the response mixture analysis, which show the emergence of a pattern of results consistent with those seen in the empirical literature regarding item parameter heterogeneity in relation to slow versus fast response times. Second, we show how the same simulated data produce relationships between RARs and RTRs that reflect the various empirical patterns documented by De Boeck and Rijmen (2020).

### 3.1. Response Mixture Analysis Simulation Results

**Figure 4:**
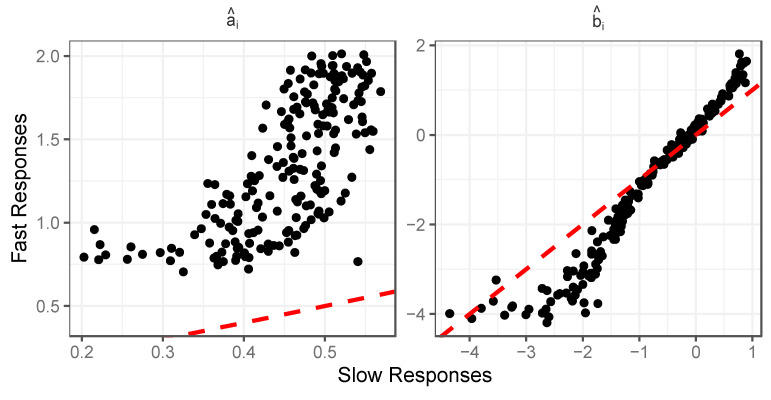
Heterogeneity of item parameter estimates in terms of discrimination (${\hat{a}}_{i}$) and difficulty (${\hat{b}}_{i}$) across fast and slow response classes; simulation illustration using 200 items and 10,000 examinees.

Figure 4 displays the results observed in terms of estimated item difficulty and discrimination in the slow and fast classes. Consistent with Molenaar and De Boeck (2018), we see that items have consistently higher discrimination estimates in the fast class and consistently lower discrimination estimates in the slow class. Interestingly, this occurs even though, in the simulation, there is no relationship between response speed and the generation of item discrimination—responses from all respondents were generated using the same item discrimination parameter. This result is not a scaling artifact, as we have a common-person design, in which the same respondents (having the same θ) provide the ability to link IRT metrics across classes. In terms of item difficulty, we also see effects that closely resemble those observed in published empirical analyses. For more difficult items, the fast response class displays higher difficulty estimates than the slow class; for easier items, the fast response class shows lower difficulty estimates than the slow class. This again is observed despite simulating the responses for all examinees using the same item difficulty parameters.

**Figure 5:**
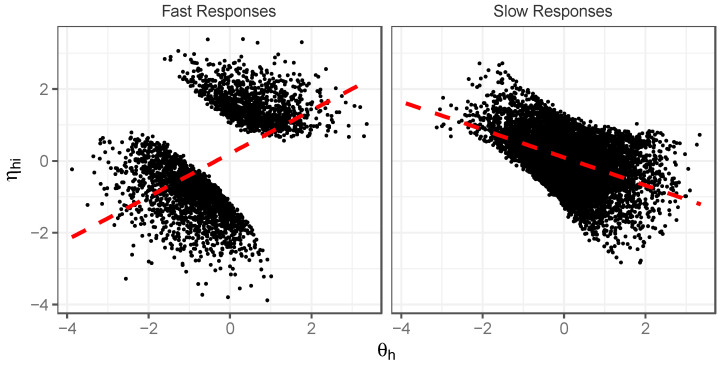
Scatterplots of ${\theta_{h},\eta }_{hi}$ distributions for fast and slow responses to medium-difficulty item (b=−0.00); simulation illustration.

Since this is a simulation, we can evaluate why these patterns of effects emerge by examining the conditional distributions of θ and $\eta_{i}$ seen in the fast and slow classes. Figure 5 displays a scatterplot of the generated θ and $\eta_{i}$ parameters for the fast and slow response classes, respectively, for a medium-difficulty item (b approx. 0). Note that for responses to this item that are slow, we have a bivariate distribution for $\theta ,\eta_{i}$ that has a strong negative correlation (as reflected by the red line)—respondents that have high θ have lower $\eta_{i}$, and those with lower θ have higher $\eta_{i}$. For fast responses, the opposite occurs: respondents with high θ have high $\eta_{i}$, and those with low θ have low $\eta_{i}$. As a result, we see a positive correlation between θ and $\eta_{i}$ (as reflected by the corresponding red line) in the fast class. Recalling that our IRT analysis producing these heterogeneous discrimination estimates is only attending to the effects of θ, it then becomes clear why the estimated item discrimination is low in the slow class but high in the fast class. The effects of θ are ultimately being exaggerated in the fast class (as effects related to $\eta_{i}$ are being attributed to θ) but are understated in the slow response class (effects related to $\eta_{i}$ are working in the opposite direction to the effects of θ).

**Figure 6:**
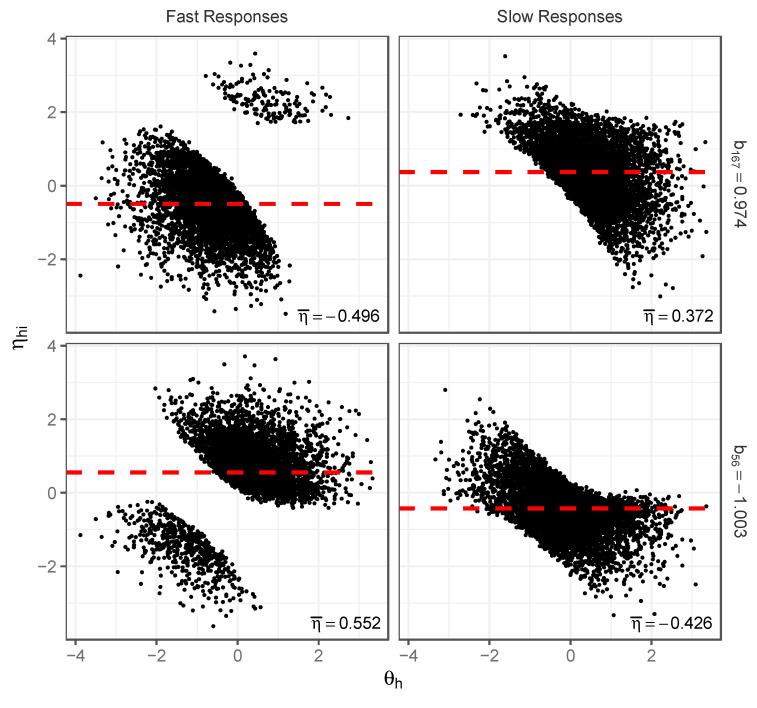
Scatterplots of ${\theta_{h},\eta }_{hi}$ distributions for fast and slow responses and their associated mean $\eta_{i}$ estimates for difficult item (Item 167, $b_{167}=0.974$, top two figures) and easy item (Item 56, $b_{56}=-1.003$, bottom two figures); simulation illustration.

A related phenomenon explains the differences in difficulty estimates between classes. Figure 6 shows scatterplots of ${\theta_{h},\eta }_{hi}$ for fast and slow responses to a more difficult item (top two figures) and to an easier item (bottom two figures). Relative to the medium-difficulty item in Figure 5, note how the true item difficulty difference has altered the scatterplots of both the slow and fast responses. For the fast responses, the item with a greater difficulty now has a larger proportion of points in the lower left of the plot (representing fast incorrect responses), while the item with lesser difficulty has a larger proportion in the upper left of the plot (representing fast correct responses). The bivariate distributions for slow responses also shift as a consequence. Most important to the observed change in difficulty estimates across classes are the mean levels of $\eta_{i}$ seen from the scatterplots of the fast and slow responses. As $\eta_{i}$ is not accounted for in the 2PL analysis, these mean differences are absorbed into the item parameter estimates for each class (Lyu et al. 2023). In the fast class, the mean $\eta_{i}$ is negative on the difficult item (making the difficulty estimates of the 2PL analysis increase); for the easier item the mean $\eta_{i}$ is positive, making the b estimate decrease. The exact opposite effects occur for the slow class. Importantly, these differences across all classes occur despite no differences in the generating item parameters for any of the respondents.

Besides replicating the findings of these prior empirical studies, our simulation results also speak to the *interpretation* of these findings from our alternative modeling perspective. In our simulation, the generating item’s difficulty and the discrimination parameters of the item are exactly the same for all respondents; the systematic differences we observe in the item parameter estimates for the slow and fast response classes are only due to the different conditional distributions of $\eta_{i}$ expected for the fast versus slow response classes. These differences are absorbed into the estimated item parameters of the two classes (see Lyu et al. 2023). Thus, from the perspective of our model, the differences seen in item parameter estimates would be seen more as psychometric artifacts due to these unaccounted-for distributional differences in $\eta_{i}$ in the subpopulations of respondents classified into fast and slow classes for each item; they do not reflect a greater or lesser role of θ in the respondent’s performance on the item, nor do they imply any difference in the psychological response process for respondents providing fast versus slow responses. We think of the effects as “artifacts” here, in the sense that under this scenario the interpretations given to item parameter differences, that they are changes in measurement, are actually differences in the features of the respondents in each class. We provide a further investigation into the relationships between the response mixture parameter estimates and parameters of our conceptual model in the Supplementary Materials Part II.

### 3.2. Simulation Results That Replicate the RAR/RTR Patterns in De Boeck and Rijmen (2020)

Other features consistent with empirical data observations also emerge from this same simulation analysis. A recent review by De Boeck and Rijmen (2020) highlighted several features of RAR and RTR associations that have emerged in empirical studies and that Kang et al. (2022) show can be explained by introducing person–item interactions into the D-diffusion IRT model. These include (1) non-zero dependencies between the RAR and RTR, (2) a positive relationship between item difficulty and RAR/RTR correlations, (3) associations between latent proficiency and RAR/RTR correlations, and (4) curvilinearity in RAR/RTR dependencies. As we seek to show below, each of these effects can be appreciated as manifestations of the presence of item-specific factors and the response time model introduced by our simulation. For these analyses, we rely on the exact same large, simulated dataset considered in the previous analysis.

**Figure 7:**
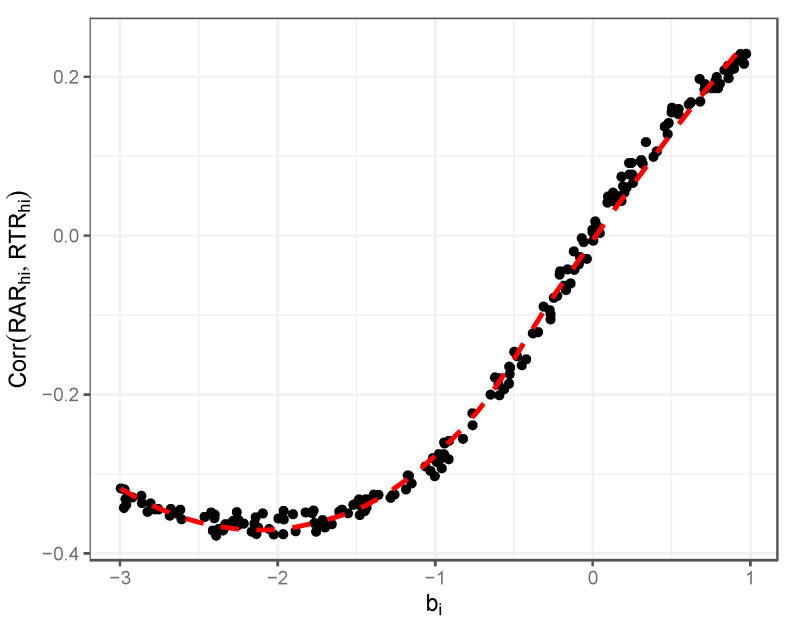
Illustration of correlation between item difficulty and estimated response accuracy/response time residual correlations; simulation illustration.

Figure 7 illustrates results relevant to the first two empirical observations above. Figure 7 shows the estimated correlation between the RAR and the response time residual in relation to item difficulty (note that, in this case, the response time residual is essentially just the variability in response time, $T_{hi}$, since we did not add in respondent or item effects related to response time, or any additional residual time effects). As discussed by De Boeck and Rijmen (2020), a robust empirical finding is the tendency to see an increasingly positive correlation between the RAR and RTR as item difficulty increases. In our simulated data, we see the same relationship. For items that are easy, there are negative associations with the response accuracy residual, but the correlations consistently move in a positive direction as the difficulty increases.

**Figure 8:**
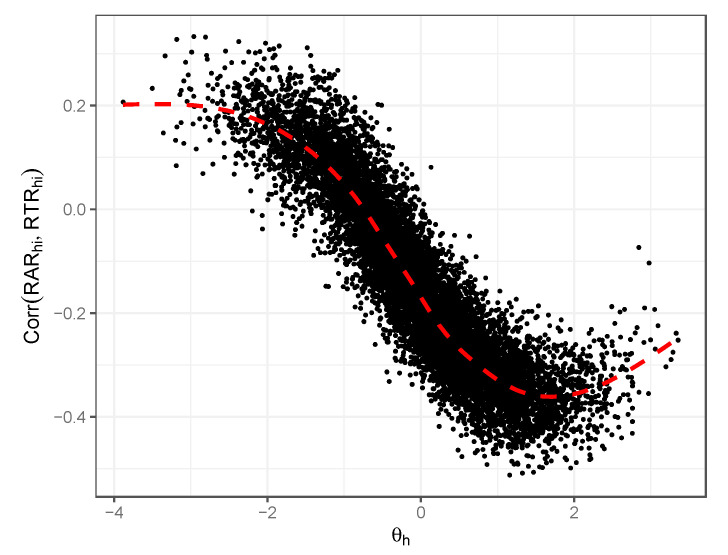
Illustration of relationship between respondents’ general latent proficiency and estimated response accuracy/response time residual correlations; simulation illustration.

We can also consider the empirical associations seen between the latent proficiency θ and RAR/RTR associations as the empirical observation (3) above. Figure 8 displays these associations as estimated from our simulated data. Figure 8 shows the true generating θ as the *x*-axis and the estimated correlation between RAR and RTR at the respondent’s level in our simulation. As we simulated 200 item responses per respondent, each of these respondent-level correlations is estimated from 200 observations. Again, as discussed by De Boeck and Rijmen (2020), we see a strong negative correlation between proficiency level and this residual correlation, an additional empirical pattern they highlight.

**Figure 9:**
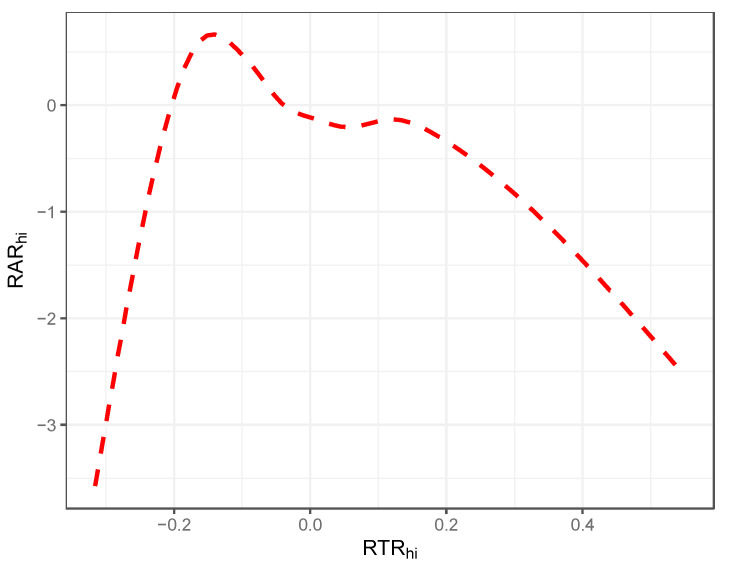
Illustration of smoothed functional relationship between RTR and RAR; simulation illustration.

Our last example concerns the curvilinear relationship seen between response time and response accuracy. Figure 9 displays a smooth function estimate of the RAR against the RTR (*x*-axis). Here, too, our pattern corresponds closely to the empirical observations observed in Chen et al. (2018) and Bolsinova and Molenaar (2018) and reported by De Boeck and Rijmen (2020)—specifically, those which state that at low levels of RTR, the relationship increases up to a point below 0, where it then begins to decline such that, for the majority of RTR observations, its relationship with RAR is negative.

Taken together, we observe the ease with which our simple psychometric perspective, which appeals to the presence of item-specific factors, can produce the same patterns of dependence between RARs and RTRs as seen in the empirical literature and discussed by De Boeck and Rijmen (2020). The strengths of the patterns seen in our simulation results are strong. They may be weakened in actual data due to other factors. In the Supplementary Materials Part I, we consider some additional simulation conditions in which we (1) manipulate the strength of item-specific factors by modifying their variance and (2) incorporate the main effects associated with persons and items in relation to response time, as well as additional response time residual variances. As seen in the Supplementary Materials, such factors can play a role in explaining why RAR-RTR patterns are not always as strong as seen in our simulations.

## 4. Discussion

In this paper, we presented a psychometric perspective on joint models of response time and response accuracy that emphasize aspects of the response accuracy residual as exogenous and predictive of response time. This perspective builds on the notion of item-specific factors (Lyu et al. 2023) as having a simultaneous influence on both response accuracy and response time. This is a new perspective to those considered by Bolsinova et al. (2017a), but one that would seem highly plausible for many cognitive achievement tests. Although admittedly less interesting from a psychological perspective, we believe this psychometric perspective is important, as the true causes of the association between response accuracy and response time may vary from one test setting to another. Adding our perspective to those previously presented encourages investigators to think carefully about different possible interpretations that can be given to explain the relationships between response accuracies and response times.

As noted, these findings are not entirely new, as our psychometric model essentially represents a perspective that can be taken on the D-diffusion IRT model with person–item interactions presented by Kang et al. (2023). However, our approach provides an explanation for these interactions, an explanation found all the way back in the original conceptualization of the common factor model. We believe this perspective is often lost in a rush to assume that latent proficiency represents the only relevant characteristic of a person brought to their experiences of items on a test.

Importantly, our perspective provides a very different interpretation of the heterogeneity of IRT item parameter estimates seen in relation to response times in empirical studies. Specifically, under our psychometric perspective, the observed item parameter estimate heterogeneity would be viewed more as a psychometric artifact, as both the fast and slow responses are in actuality equivalently affected by general latent proficiency. There is no difference in the response process introduced in our simulation. As components of the response accuracy residuals, item-specific factors are orthogonal to general latent proficiency. While Lyu et al.’s (2023) paper considers the role of item-specific factors in the broader context of sequential and IRTree models, in this paper we suggest their relevance also in providing a different perspective on response mixture analyses, which are sometimes represented using IRTrees (e.g., DiTrapani et al. 2016). Of course, we hasten to add that these conclusions solely follow from our conceptual model; alternative models that choose not to assume an influence of the RAR on response time would naturally not reach this conclusion, and there is no way to definitely prove that our perspective is right or wrong in comparison to these alternative perspectives.

As noted by a reviewer, it may also be possible to take a trait-like perspective on aspects of the RTR that impact the RAR, leading to a model similar to Bolsinova et al. (2017a)’s Figure 1 Model C. Such a perspective would emphasize the item-specific factor as a component of the RTR. We chose to emphasize the item-specific factor as a component of the RAR largely because the presence of such factors has long been a part of psychometric theory, particularly in relation to the common factor model. But it is certainly conceivable that in certain contexts one might prefer to think in the reverse direction.

As empirically it will likely be difficult to establish directionality between response accuracy and time, theory is likely the best guide as to which perspective may be most useful to take. We presented examples earlier in the paper that we think likely conform to settings where the effects primarily flow from response accuracy to response time. In the context of an example conceptual item, for example, it is conceivable that rapid incorrect answers to the item could have been improved by students spending more time on the item, but the more likely scenario seems that such responses reflect students not knowing the answer and consequently making a rapid guess about the item as a result of their lack of knowledge.

It is of course also possible that both the phenomenon we consider and true response process heterogeneity co-exist; namely, that the empirical heterogeneity in item parameter estimates is in part a manifestation of different psychological response processes and in part a psychometric artifact. It could also be the case that a different form of true variability in the psychological response process is present, that the phenomenon we present in this paper is also present, and that the combination of their effects produces a different type of pattern in the item parameter estimates that reflects their combined influences. In our opinion, however, the seemingly close correspondence between our simulation result estimates and the effects seen in the empirical analysis seem more than coincidental. The fundamental difference in perspectives seems driven by whether one thinks more in terms of effects in the direction of response times to accuracy or in the direction of accuracy to response times.

While, in this paper, we advance our alternative perspective as having implications for interpreting item parameter estimate heterogeneity, we believe the implications of our perspective may also extend beyond this. Our alternative model seemingly also has implications for efforts toward relating the RTR to latent proficiency through cross-loadings (Bolsinova and Tijmstra 2018). In particular, the presence of an interaction would naturally complicate efforts to do this. An ability to understand how the RTR might inform proficiency seemingly requires knowledge of the proficiency or RAR, neither of which is known. We think that the question of how (or whether) the presence of this interaction should affect the joint modeling of response accuracy and response time (or alternatively, the implications of ignoring the interaction and using residual response times to inform the measurement of latent proficiency) could be worthy of consideration. Additional attention could also be given to how other psychometric characteristics of the test (e.g., the distribution of item difficulties) or testing conditions (e.g., the nature of time constraints or student motivation) might affect the patterns anticipated under the model presented. As our objective in this paper was mainly one seeking to justify the psychometric perspective as one that can explain some of the consistent empirical observations in the literature, we leave these investigations to future studies.

## Data Availability

The dataset presented in this article is not readily available as it is owned by the Testing and Evaluation Center at the University of Wisconsin, Madison, and was made available to the authors for the analysis shown in this article. Requests to access the dataset should be directed to Sonya Sedivy (ssedivy@wisc.edu).

## Supplementary Materials

The following supporting information can be downloaded at: https://www.mdpi.com/article/10.3390/jintelligence12080074/s1. Additional materials related to this paper, including additional simulation illustrations and R code, can be accessed at https://uwmadison.box.com/s/og2d39y2wkjdrfgc0gdltofo4pfpisdp, accessed on 24 July 2024.
