# Supplementary material for: A Psychometric Perspective on the Associations between Response Accuracy and Response Time Residuals
Source: J Intell. 2024 Jul 31;12(8):74. doi: 10.3390/jintelligence12080074 (PMC11355612; doi:10.3390/jintelligence12080074)
Supplement: Supplementary file 1 [file jintelligence-12-00074-s001.zip › supplement_part2.pdf]

Supplementary Material for "A Psychometric Perspective on Associations between Response Accuracy and Response Time Residuals", Part II

We conducted additional analyses based on our simulation to lend further insight into the relationships between generating parameters of the psychometric model presented in the paper and the estimates returned from the mixture response analysis. Our simulation can be replicated using the R code provided for readers who are interested in exploring other aspects of these parameters and mixture response estimation results. We acknowledge that these relationships can be somewhat complex; nevertheless the results below can help lend additional insight into findings that also appear to support the interpretations given in the paper.

First, we add to the figures in the paper by showing the relationship between the generating parameters (a's and b's) in the simulation illustration, and the corresponding estimates in the response mixture analysis:

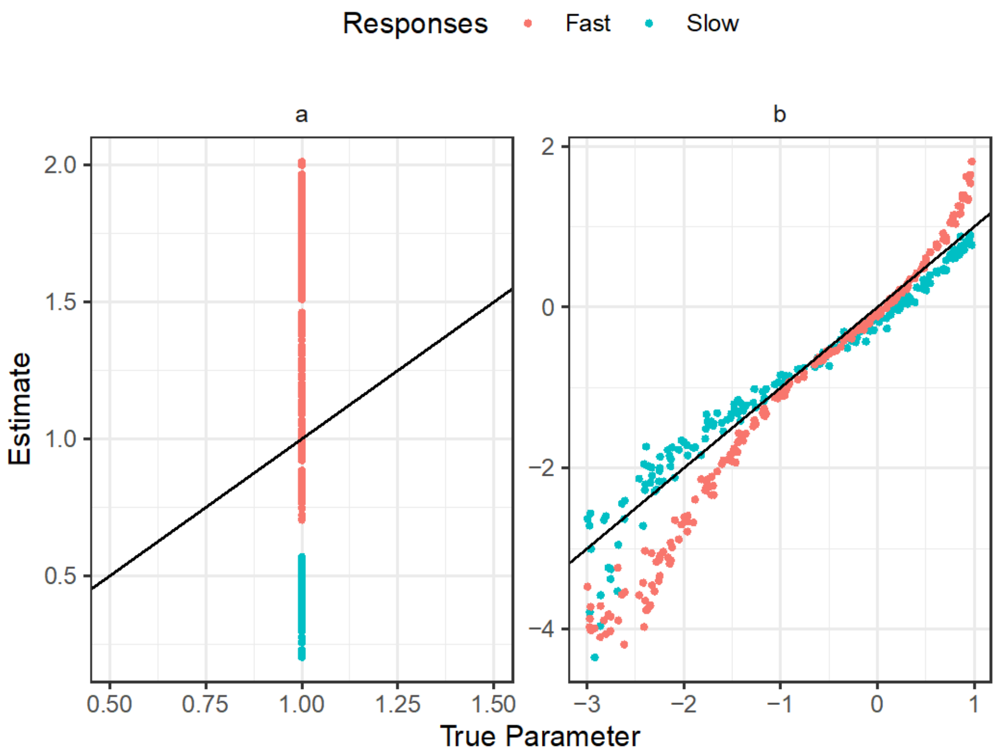

Figure 1''. Relationships between Generating Item Parameters and Mixture Response Item Parameter Estimates, Fast and Slow Classes, Simulation Illustration.

As shown in Figure 1′′ , we are able to see how the estimates depart from the generating parameters, and can also more easily see the "bias" in the response mixture item parameter estimates ("bias," of course, only if assuming the psychometric perspective presented in the paper is correct) in relation to the true generating parameter. For the a's, as noted in the original paper, we generated all of the items to have a value of 1. As a result, while we can see the bias through the plot, we do not have the ability to see how/whether that bias changes across levels of a, as there was no manipulation of that generating parameter. However, for the bs, we see a pattern of values consistent with that seen in Figure 4 within the paper, now implying a reverse directionality to the b estimate bias according to whether the responses are fast or slow.

Second, we can examine relationships between distributional characteristics of $\eta_{hi}$and the item parameter estimates within the respective mixture response analysis classes. Figure 2′′ shows the relationship between the item parameter estimates for each class and the mean(η_{hi} | class). Each point represents a different item for a class. We think the results related to the b-parameter estimates are most interesting, and conform to the figure shown for the b estimates across classes in Figure 1′′. Basically, for slow responses, the item difficulty estimates get progressively higher as the mean(η_{hi} | class) increases, while for fast responses, we have a nonmontone relationship between mean(η_{hi} | class) and the item difficulty estimate. This nonmonotonicity appears to be driven by the fact that the fast responses reflect two types of behaviors (i.e., quick guesses and immediate correct responses), where the relative allocation of each response type depends on the difficulty of the item. For the a estimates, the more interesting result is seen in the fast class. The curvilinear relationship seen is related to the effect seen for the b's. The a estimates are greatest in the fastest class when η_{hi} is near 0 because this often corresponds to conditions where there are an approximately equal number of fast responses due to rapid guessing and immediate corrects, making the discrimination most exaggerated due to the positive correlation between theta and eta in the fast class (see plot in Figure 3′′ for what we think is a better characterization of what is happening with the a's.)

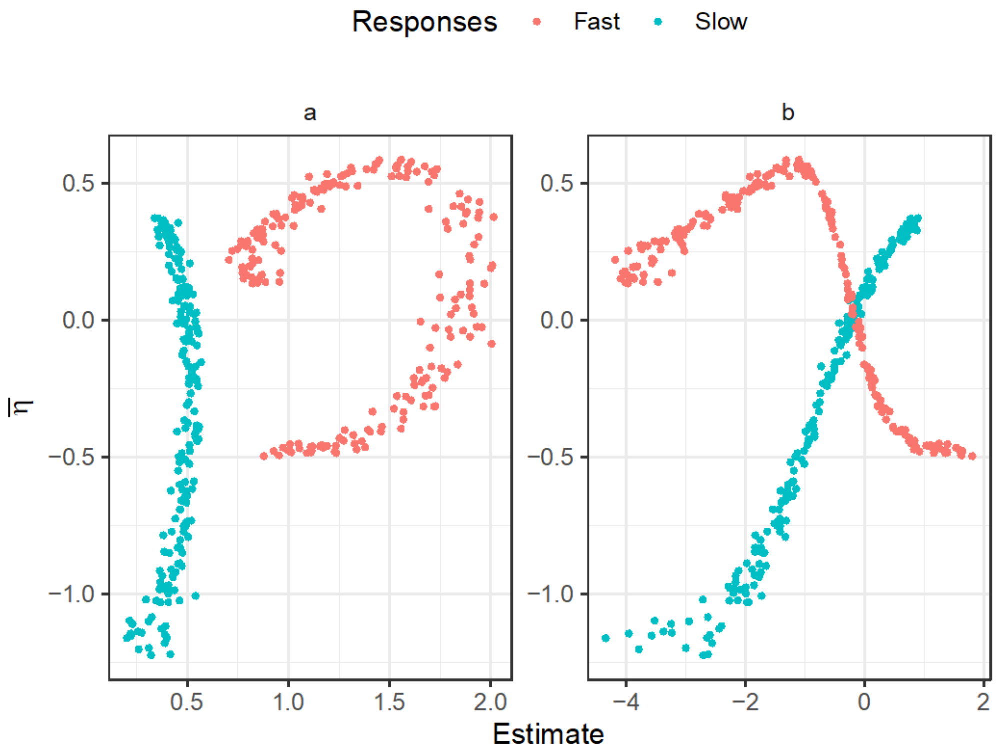

Figure 2′′. Relationships between Mixture Response Item Parameter Estimates and Mean(η_{hi} | class), Fast and Slow Classes, Simulation Illustration.

Figure 3′′ presents the relationship between the correlation between η_{hi} and θ in each class and the mixture response item parameter estimates. We look at this because of its anticipated relationship to the a parameter estimates especially. The left figure below confirms our anticipated effect. The slow class a's are low because there is a consistently negative correlation between η_{hi} and θ in the slow class, while the fast class a's are higher because of the more positive relationship between η_{hi} and θ in the fast class. The correlation clearly also affects the b's in each class, especially the fast class, but is again a function of the two ways in which fast responses occur, which induces the nonmontone relationship.

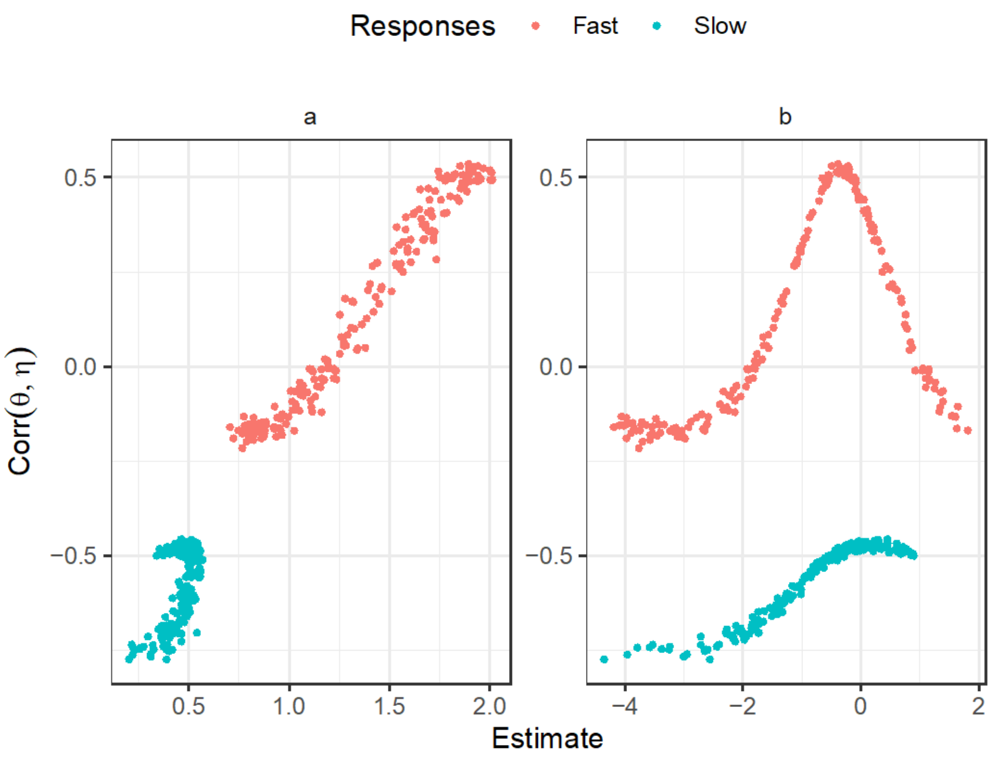

Figure 3′′. Relationships between Mixture Response Item Parameter Estimates and Correlation between η_{hi} and θ, Fast and Slow Classes, Simulation Illustration.

There are naturally additional issues that could be further studied, and we encourage readers to use our R code to study these issues further, as well as to manipulate our simulation conditions (e.g., the generating item parameters) to see how that might further affect these observations. From our perspective, there can be significant complexity to these relationships, and it can often be difficult to look at relationships between two features without attending to other features that play a role. But we hope the observations above add to reader understandings of what is occurring in our simulation.
