# Supplementary material for: A Psychometric Perspective on the Associations between Response Accuracy and Response Time Residuals
Source: J Intell. 2024 Jul 31;12(8):74. doi: 10.3390/jintelligence12080074 (PMC11355612; doi:10.3390/jintelligence12080074)
Supplement: Supplementary file 1 [file jintelligence-12-00074-s001.zip › supplement_part1.pdf]

Supplementary Material for "A Psychometric Perspective on Associations between Response Accuracy and Response Time Residuals", Part I

Here we show more simulation results under other scenarios. They generally follow the same pattern as those shown in the manuscript, suggesting that our findings are consistent and robust across different settings.

1 Changing the Variances of η_{hi} and $\varepsilon_{U_{hi}}$

In this section, we consider three scenarios: $\mathrm{Var}(\eta_{hi}) = 0.5, 1, 2$. Under each scenario, we fix $\mathrm{Var}(\theta_h) = 1$ and let $\mathrm{Var}(\varepsilon_{U_{hi}}) = \frac{\pi^2}{3}\mathrm{Var}(\eta_{hi})$. Figures 4, 7, 8 and 9 in the manuscript are redrawn as Figures 4′, 7′, 8′ and 9′.

2 Adding Variations to T_{hi}

In this section, we add respondent level speedness parameters to the response time. Now the response time follows

$$T_{hi} = \alpha_i(\beta_i - \tau_h) + P(U_{hi} = 1 \mid \theta_h + \eta_{hi} - b_i) - 0.5 + \varepsilon_{T_{hi}},$$

where

$$\begin{aligned}(\theta_h, \tau_h)^\mathsf{T} &\sim \mathcal{N}\left(\begin{bmatrix}0\\0\end{bmatrix}, \begin{bmatrix}1 & 0.3\\0.3 & 1\end{bmatrix}\right),\\ \alpha_i &= 1,\\ \beta_i &\sim \mathcal{U}(-1, 1),\\ \varepsilon_{T_{hi}} &\sim \mathcal{N}(0, 0.5^2).\end{aligned}$$

Figures 4, 7, 8 and 9 in the manuscript are redrawn as Figures 4″, 7″, 8″ and 9″.

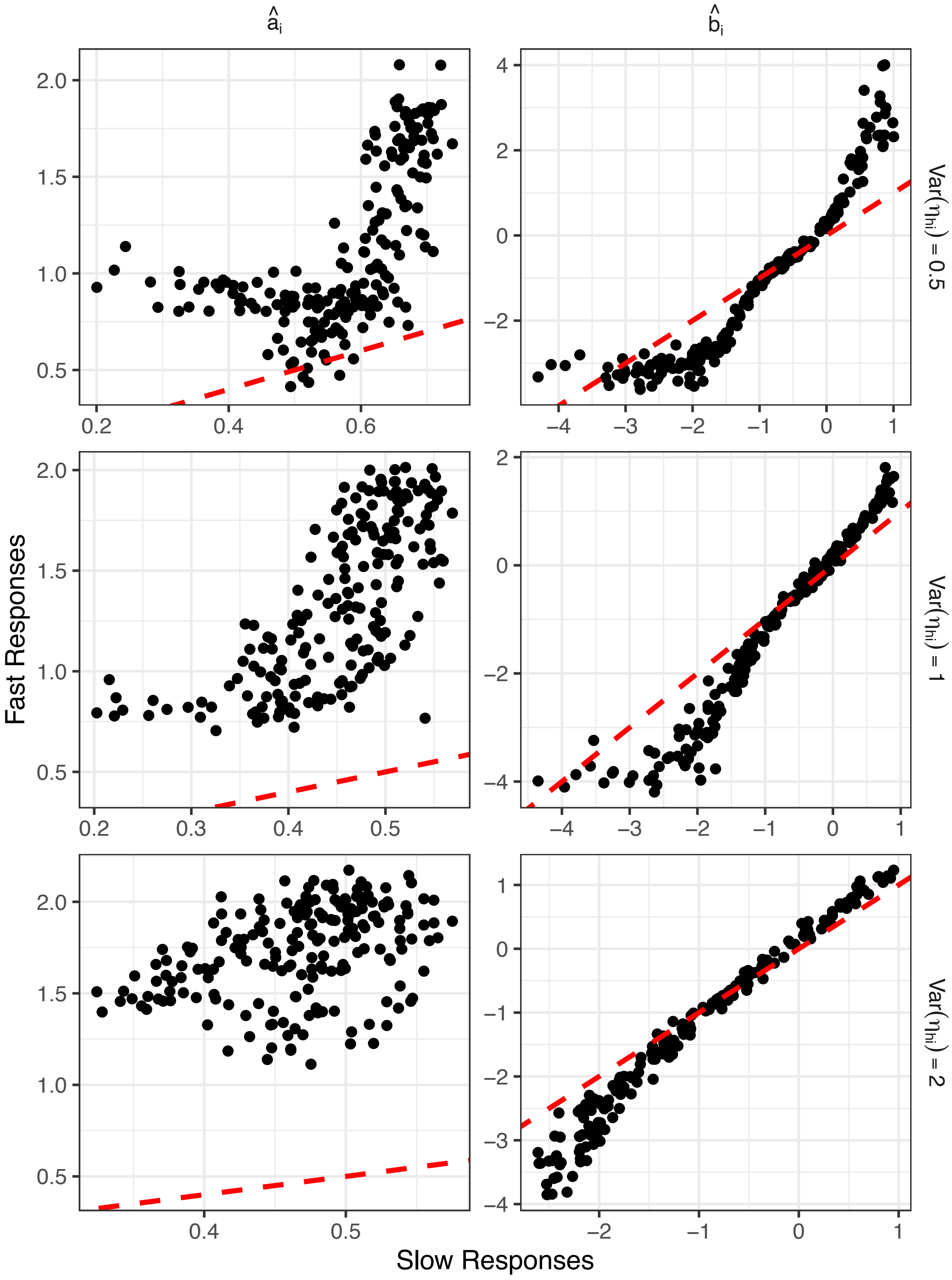

Figure 4′: Item Parameter Estimate Heterogeneity, for Discrimination ($\hat{a}_i$) and Difficulty ($\hat{b}_i$) Across Fast and Slow Response Classes, Simulation Illustration, 200 Items, 10000 Examinees

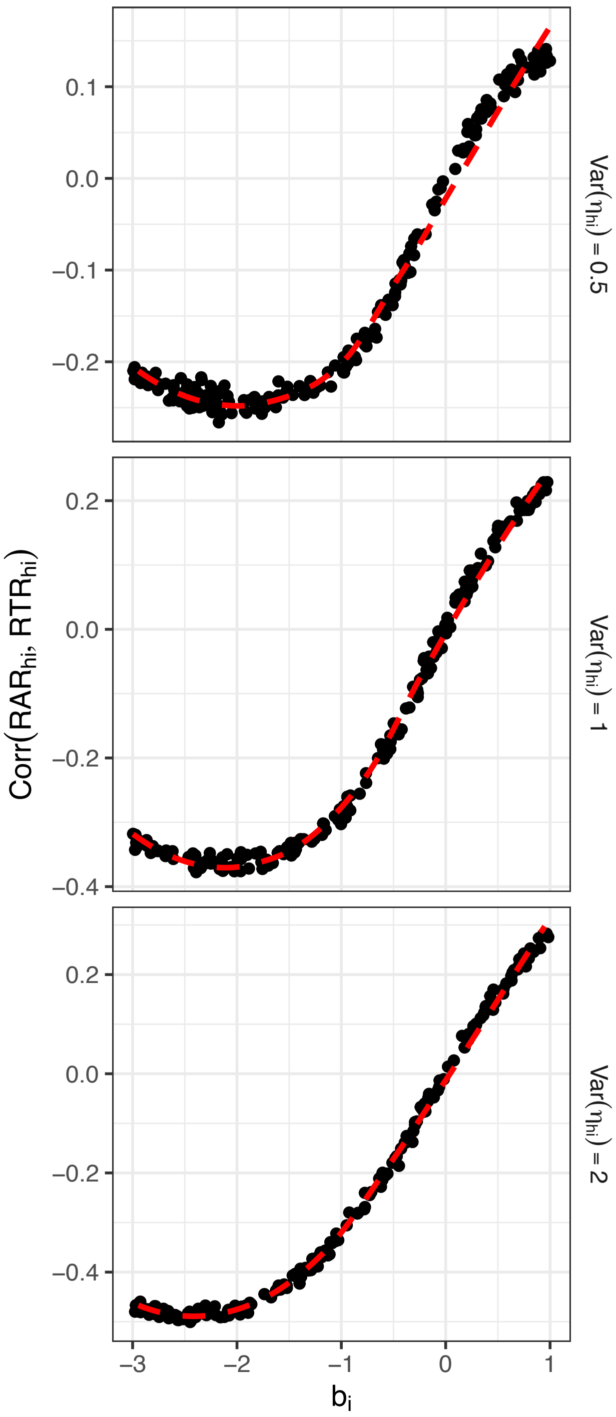

Figure 7′: Illustration of Correlation Between Item Difficulty and Estimated Response Accuracy/Response Time Residual Correlations, Simulation Illustration

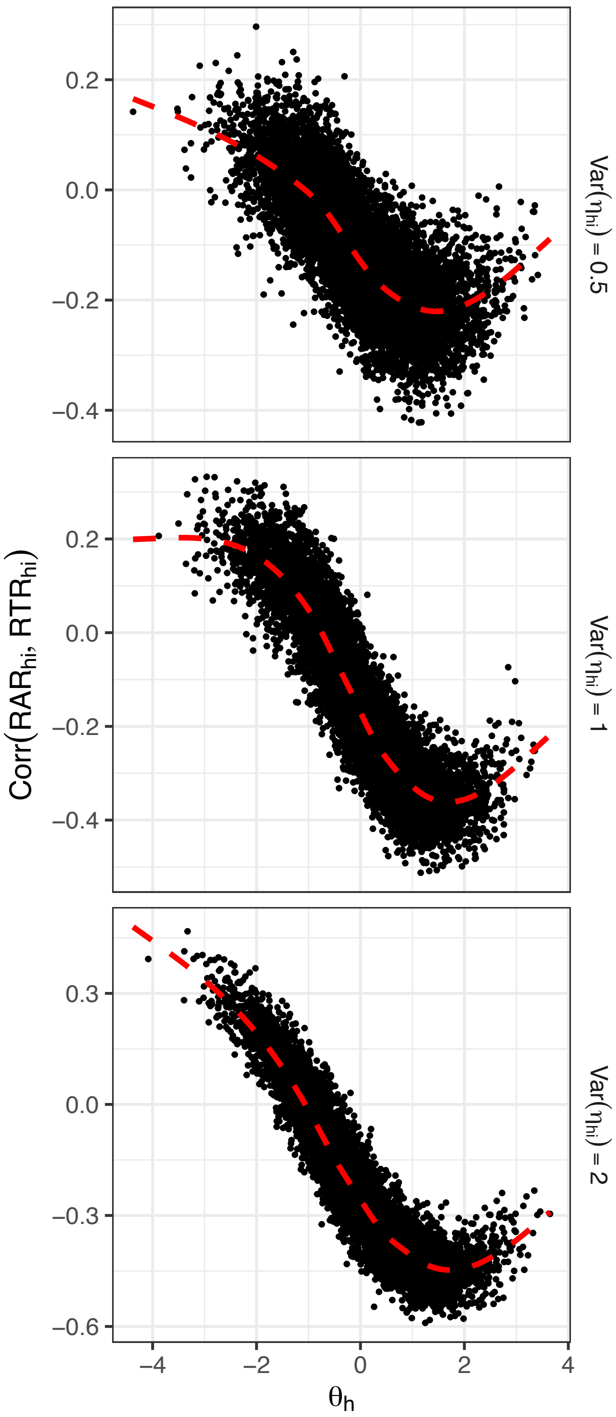

Figure 8′: Illustration of Relationship Between Respondent Theta and Estimated Response Accuracy/Response Time Residual Correlations, Simulation Illustration

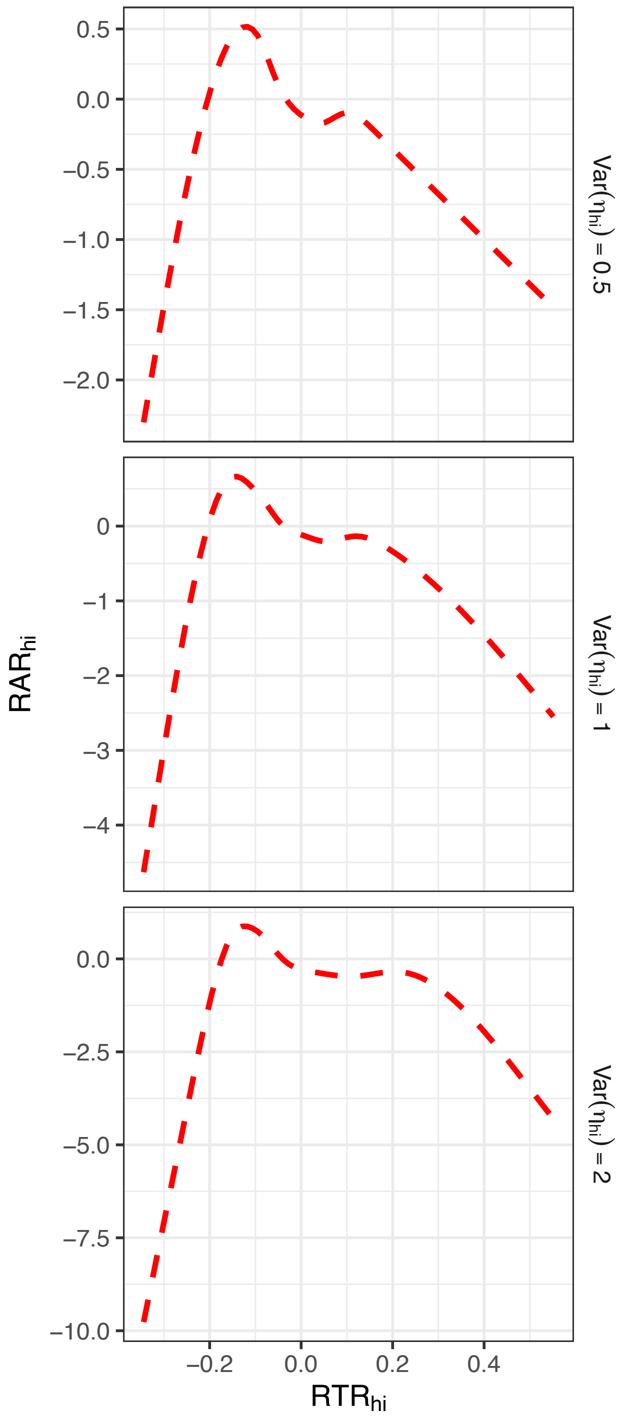

Figure 9′: Illustration of Smoothed Functional Relationship Between RTR and RAR, Simulation Illustration

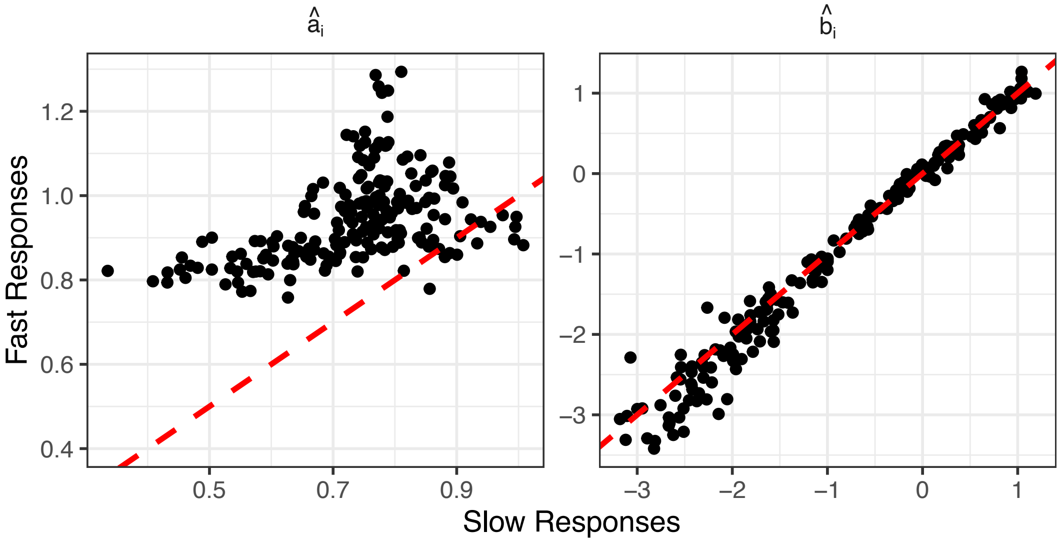

Figure 4″: Item Parameter Estimate Heterogeneity, for Discrimination ($\hat{a}_i$) and Difficulty ($\hat{b}_i$) Across Fast and Slow Response Classes, Simulation Illustration, 200 Items, 10000 Examinees

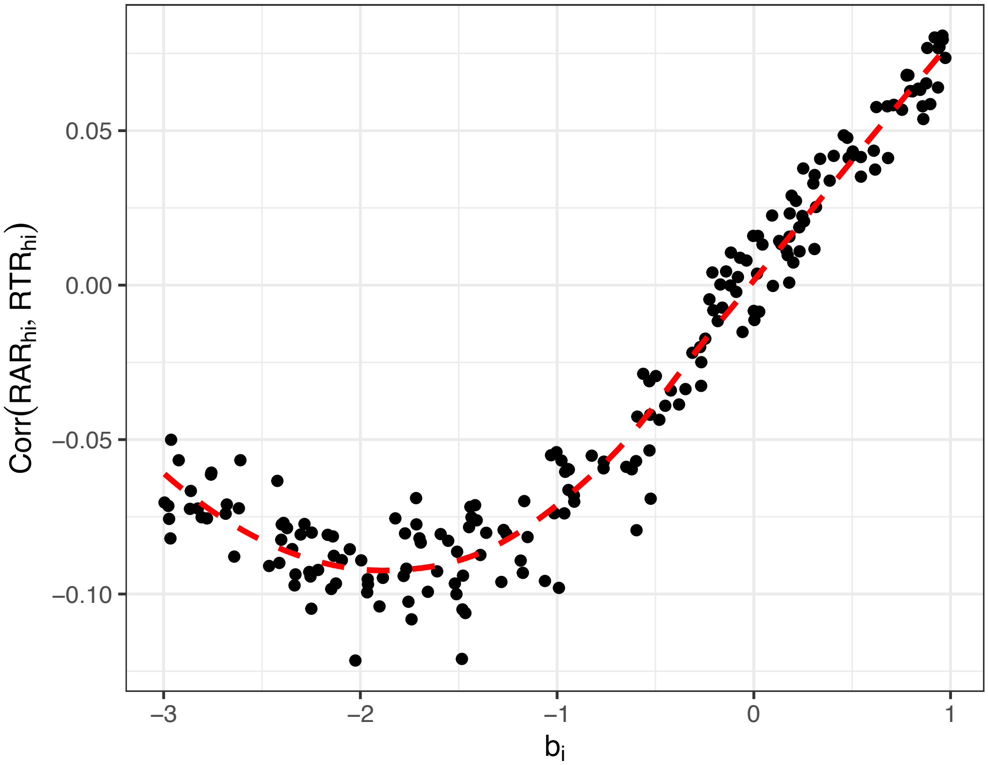

Figure 7″: Illustration of Correlation Between Item Difficulty and Estimated Response Accuracy/Response Time Residual Correlations, Simulation Illustration

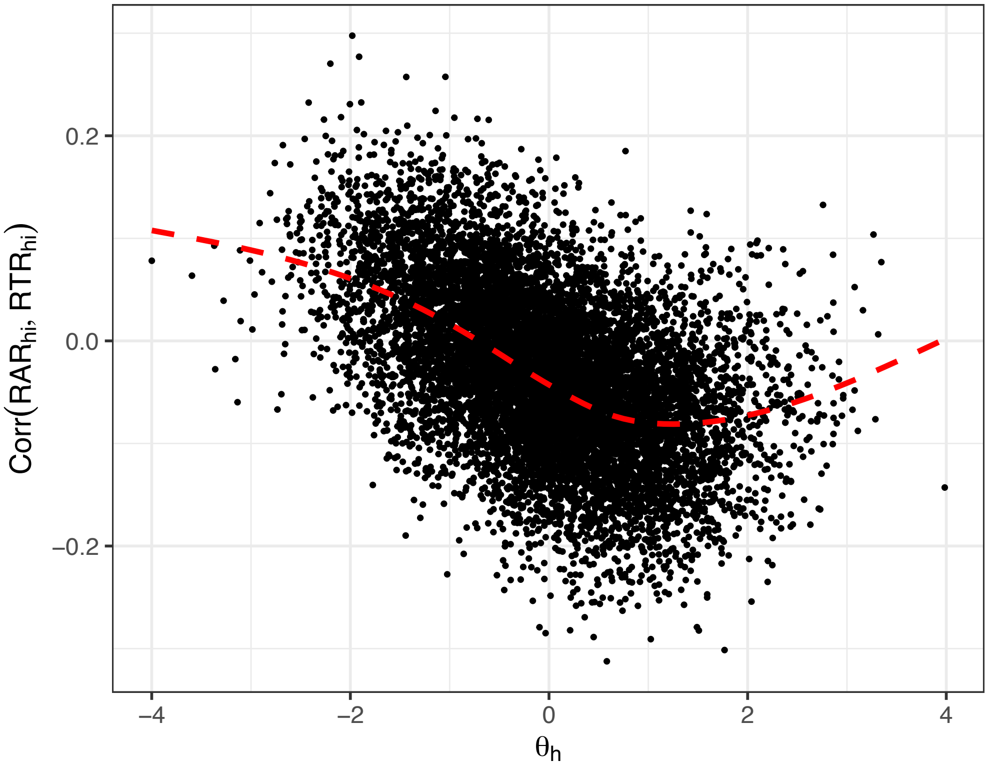

Figure 8″: Illustration of Relationship Between Respondent Theta and Estimated Response Accuracy/Response Time Residual Correlations, Simulation Illustration

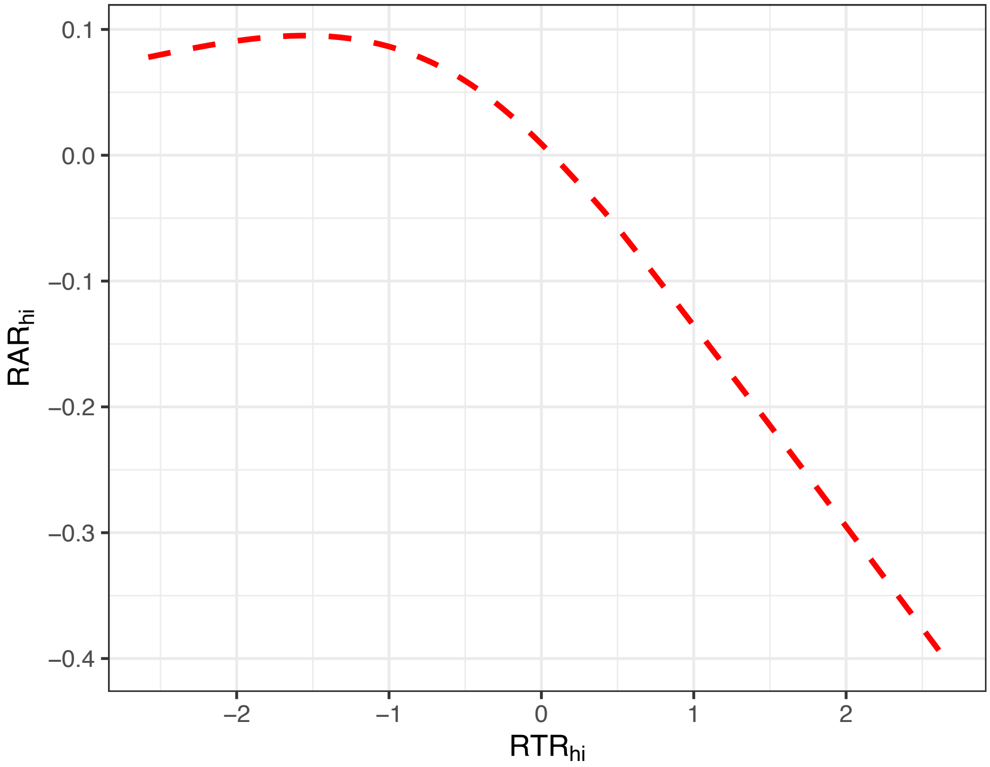

Figure 9′′: Illustration of Smoothed Functional Relationship Between RTR and RAR, Simulation Illustration
